# Exploring climate-related health risks and behaviors among vulnerable populations in the eastern Mediterranean: the cases of Jewish and Bedouin Negev desert residents

**DOI:** 10.3389/fpubh.2026.1892365

**Published:** 2026-09-10

**Authors:** Anat Rosenthal, Maya Negev, Matan E. Singer, Soha Natour, Irit Porat, Stav Shapira

**Affiliations:** 1 Department of Health Policy and Management, School of Public Health, Faculty of Health Sciences, Ben-Gurion University of the Negev, Be'er Sheva, Israel; 2 School of Public Health, Faculty of Health and Welfare Sciences, University of Haifa, Haifa, Israel; 3 Haifa Center on the Politics of Inequality, University of Haifa, Haifa, Israel; 4 Department of Public Policy and Administration, Guilford Glazer Faculty of Management, Ben-Gurion University of the Negev, Be'er Sheva, Israel; 5 School of Public Health, Faculty of Health Sciences, Ben-Gurion University of the Negev, Be'er Sheva, Israel

**Keywords:** Bedouin, climate change, coping mechanisms, desert, everyday adaptation, severe weather events, vulnerable populations

## Abstract

**Introduction:**

The Negev region of Israel is susceptible to the impact of climate change, and future predictions include an increase in the intensity, length, and frequency of extreme weather events. The Negev is also home to a variety of vulnerable communities characterized by low socioeconomic status and inadequate availability of infrastructure as well as inadequate access to healthcare services. In this article we explored climate-related risks and coping practices of Jewish and Bedouin members of vulnerable communities in the Negev.

**Methods:**

The article draws from 10 focus groups (n = 69 participants) of vulnerable Jewish and Bedouin residents of the Negev, conducted between March and August 2023.

**Findings:**

We identified coping mechanisms at the individual and community level, and at the level of the state and its institutions, with everyday adaptations developed by communities where the state is absent. Vulnerable populations’ experiences of climate change are impacted by factors such as poverty, social and political marginality, violence, and barriers in accessing health and other services.

**Discussion:**

We argue that the impact of climate change is experienced in the context of other vulnerabilities and exacerbated by them. Climate change effects are not a stand-alone phenomenon, and climate change adaptation and mitigation strategies cannot be distinguished from other challenges impacting the health and well-being of populations.

## Highlights

- Vulnerable and marginalized populations are disproportionately exposed to the impact of climate change.
- The impact of climate change is experienced in the context of other vulnerabilities such as poverty and political marginality.
- In the desert, vulnerable populations are especially exposed to extreme heat, dust storms, and floods, and have fewer means to protect themselves.
- There are no “one-size-fits-all” climate interventions for vulnerable populations in different geographic and cultural contexts.
- Strengthening vulnerable communities during routine times will allow for an easier mobilization of support during emergencies.

## Introduction

The Negev region of Israel is susceptible to the impact of climate change. An increase in temperature and decrease in precipitation have been observed, and future predictions include an increase in the intensity, length, and frequency of extreme weather events as a result of changing climate conditions (1–3). The Negev is also home to a variety of vulnerable communities characterized by low socioeconomic status and inadequate availability of infrastructure and access to healthcare services (4–6). The current study’s aim was to identify climate-related risks, health perceptions and needs, and associated behaviors among Jewish and Bedouin populations. Drawing from 10 focus groups (*n* = 69 participants) of vulnerable Jewish and Bedouin residents of the Negev region conducted between March and August 2023, we argue that the impact of climate change is experienced in the context of other vulnerabilities, and that coping mechanisms range from individual- and community-level to the level of the state and its institutions. Vulnerable populations’ experiences of climate change are impacted by factors such as poverty, social and political marginality, violence, and barriers in accessing health and other services. Climate change effects are thus not a stand-alone phenomenon, and climate change adaptation and mitigation strategies cannot be distinguished from other challenges impacting the health and well-being of populations.

Climate change effects, including rising temperatures and increases in extreme climate events such as heatwaves, droughts, floods, and wildfires, influence a variety of health factors including a rise in all-cause mortality and morbidity, heat stress, respiratory illness and allergies, and deteriorating mental health (7). Heatwaves are associated with a rise in cardiovascular and cerebrovascular diseases, including stroke (8, 9). High temperatures have been associated with a rise in homicide, violence, domestic violence, and suicide attempts (10–13). The increase in extreme hot days is expected to reduce physical activity, active transport (walking and cycling), and social gatherings and thereby increase obesity, diabetes, and loneliness (14).

To mitigate the health impacts of climate change, top-down climate policies and institutional adaptations are essential. These strategies involve coordinated efforts by governments and organizations to implement systemic changes, such as reducing greenhouse gas emissions and enhancing climate resilience (15). Simultaneously, bottom-up everyday adaptations by individuals and communities play a critical role in coping with the changing climate. These adaptations include practical, often informal, adjustments in daily life to manage the impacts of climate variability on domestic and occupational activities (16). For instance, people adjust their behavior during hot days to reduce heat exposure, change work practices and business structures in response to climate change, and adjust homes in various ways to help mitigate extreme heat (17). At the community level, networks often mobilize to provide mutual aid and support during extreme climate events, such as heatwaves or flooding, demonstrating local community resilience (18).

In Israel, hot temperatures and heat waves have been found to increase the risks of preterm birth (19), preeclampsia (20), stroke (21), outbreaks of West Nile fever (22), leishmaniasis (23), campylobacter among children (24), suicide (25) and snakebites (26). These trends are expected to intensify, especially in arid lands including the Negev. There is strong evidence that climate change impacts human health globally, nationally, and locally. Although there is a need for further epidemiological studies to help us better understand the relationships between climate events and specific health outcomes and populations, there is an even greater need for studies focusing on the local needs of the Negev communities and on the capacity-building of local health institutions (i.e., hospitals, primary care clinics, and family health centers) in order to develop climate resilience.

Although climate change affects the health of all, vulnerabilities to the impact of climate change are not shared equally across populations. The poor, the older adults, people living in informal settlements, people living with disabilities, people who are institutionalized, and people working outdoors have been identified as being disproportionately exposed to heightened physical and emotional stress caused by climate change and natural disasters (27–29). Existing marginalities and inequalities also expose individuals and communities to disproportional environmental risks across gender, class, and ethnicity (30).

The Lancet 2021 report on climate change and health identified the risks posed by climate change as overburdening the most vulnerable populations worldwide, to the extent that they are risking decades of achievements and advancements in health (31). Social vulnerabilities play a role not only as catalysts for greater exposure to the risks of climate change, but also as barriers to mitigation and adaptation strategies, as well as access to health services (28). No less important, adaptation and mitigation strategies that do not address the needs of vulnerable populations may actually create mechanisms of maladaptation and divert resources away from vulnerable populations (32, 33), and thus climate policies should address systemic inequalities and context-specific climate adaptation strategies (34). Therefore, there is a need to understand the unique vulnerabilities and needs of various populations, as well as the coping resources available to them in order to promote just and appropriate adaptation and mitigation strategies.

## Materials and methods

### Settings and study population

The Negev desert is widely recognized as one of Israel’s regions most vulnerable to the impact of climate change (1, 2). An increase in temperature and decrease in precipitation have been observed, and future predictions include an increase in the intensity, length, and frequency of extreme weather events such as heatwaves (35). Moreover, the Negev lies on Israel’s periphery and is home to a range of vulnerable populations. Many communities in the region are characterized by low socioeconomic status in local terms, and the Negev as a whole suffers from inadequate infrastructure and services (6). Existing gaps in the availability and accessibility of healthcare services in the Negev, as well as in additional health and economic indices, compared with other regions in Israel, are well documented (6, 36).

The Negev is home to the Bedouin, an ethnic Arab Muslim minority that constitutes one of Israel’s most vulnerable populations, ranking lowest in socioeconomic and educational indicators compared to other ethnic minority groups. The approximately 272,000 Bedouin of the Negev live in three types of settlements: an estimated 65% of them live in 11 government-planned townships and “newly-recognized” villages. The remainder live in so-called “unrecognized villages”: about 34 clusters of temporary, informal structures. The residents of these unrecognized villages have some access to healthcare services but completely lack access to basic infrastructure such as the national water supply and to electrical and communication networks, making them extremely vulnerable to climate change’s adverse impacts (4, 5).

**Figure 1:**
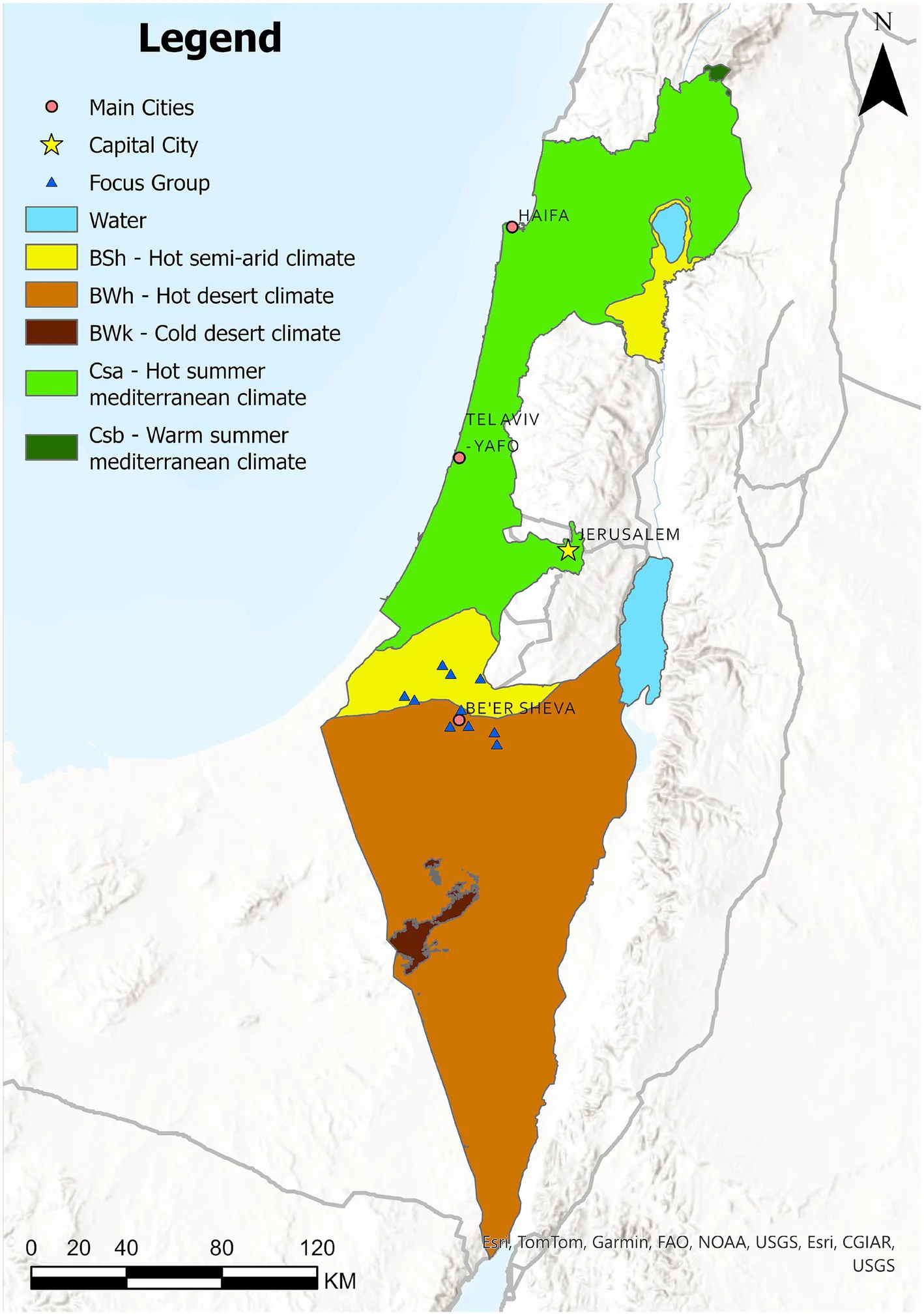
Map of study settings and climate classification (Map by authors. Representing diverse climate zones based on the Koppen Classification).

For example, in the Middle East, climate change threatens water security by lowering precipitation and changing its intensity and patterns, thereby reducing water quality and quantity, especially in sensitive regions such as the Negev, and particularly in rural Bedouin villages that are not connected to the water grid (37). Such patterns were associated with a relatively recent outbreak of leptospirosis in the North of Israel (38), thus exposing Bedouin communities to even greater health risks (see Figure 1).

### Study design

An exploratory qualitative research design was adopted, based on focus group discussions, to investigate understandings of climate-related risks, coping practices, and their experiential contexts among vulnerable Jewish and Bedouin populations in the Negev region of Israel. Focus group discussions were chosen due to their strength in promoting participants’ engagement as an instrument of data collection (39). This exploratory qualitative design included an iterative process of a preliminary codebook thematic analysis followed by reflexive revision of the codebook. The combination of strategies allowed for the development of an analytical farmwork based on the themes identified in the coding process (40, 41).

### Data collection

Ten focus groups (*n* = 69 participants) comprised of members of vulnerable Jewish and Bedouin residents of the Negev region were conducted between March and August 2023. Throughout the study, we defined vulnerabilities based on participants’ income, occupation, age, health status, living environment, and outdoor activities. Based on these defined vulnerabilities, the study team mapped potential venues for participant recruitment and approached community organizations, community groups and municipal organizations with an invitation to participate in the study highlighting its objectives. The invitation to participate in the study was made based on the characteristics of the communities (rural, unrecognized etc.) or the population (the older adults, people employed outdoors etc.).

Focus group discussion were led by trained facilitators in Arabic, Hebrew, or Russian and lasted up to 90 min. The facilitators of the focus groups were female PhD students with background in public health, climate studies and public policy. All facilitators had post-graduate level training in qualitative research methods as well as study-specific training by one of the PIs who is an anthropologist. Facilitators represented Muslim and Jewish heritage and were all residents of the region where that study was conducted. However, while being rooted in the region and having experience in research in the studied communities, they were not members of the communities sampled in the study. The facilitators knowledge of the study communities, their languages and traditions were crucial in gaining entrance to studied communities. To overcome biases that might occur due to the facilitators’ familiarity with the studied communities, the team met on a weekly basis to debrief and discuss data collection.

All the facilitators followed the same discussion guide designed specifically for the study. The discussion guide was designed based on the objectives of the study as well as on themes identified in the literature review. The guide included six modules; 1. Introduction of the study and its objectives, 2. Knowledge of climate change and its impact on health, 3. Personal experiences of climate change and associated risks, 4. Barriers and facilitators to coping with the effects of climate change, 5. Preparedness and response to climate change and severe climate events on the community and regional level, and 6. Attitudes, awareness and perceptions regarding climate change and severe climate events. Each of the modules included relevant probes and sub-questions to help guide the discussion. The guide was developed in Hebrew and subsequently translated into Arabic and Russian by native speaking team members.

Focus groups were held in cities, small- and mid-size towns, recognized and unrecognized Bedouin villages, and a kibbutz (collective community). The meeting locations were collaboratively chosen by participants and facilitators to accommodate geographical and cultural access. Therefore, meetings were held in community centers or private homes at the request of participants. As a result, focus groups in Bedouin towns and villages were conducted separately for men and women to accommodate cultural norms and traditions, ensuring a comfortable environment for open and honest discussion.

Data collection was initiated with a pre-determined list of target populations to be sampled for focus group discussions. Throughout the data collection period, the team met weekly to compare data and update the sample accordingly. A review of findings from focus group discussion, addressing known and new emerging findings, was conducted weekly in the team debrief meetings. Data collection was complete once data saturation was determined by comparing transcripts across the 10 focus groups and establishing a sufficient degree of repetition in the data (42). Due to the heterogeneous characteristics of the sample, saturation was determined for each category of locality and gender as well as for the overall sample.

### Ethics

The study was funded by a grant from the Israeli Ministry of Innovation Science and Technology (grant number 1001578050) and approved by the Ben-Gurion University Human Subject Research Committee (#380-2, dated 15/2/2023). Privacy rights of human subjects were observed, and all participants were over 18 years of age and signed a consent form. During the introduction and consent phase of the focus group discussions, participants were reminded to respect the privacy of other participants and refrain from sharing any information discussed in the meeting although complete confidentiality cannot be guaranteed. Participants were compensated at a sum of $30 per participant. Identifying information of participants was anonymized in the analysis phase of the study. Data was saved on a secure institutional server, and only team members had access to the study’s database.

### Data analysis

All 10 focus group discussions were audio-recorded and transcribed verbatim. Transcripts of focus group discussions conducted in Arabic and Russian were translated into Hebrew by native speaking team members to ensure accessibility for all members of the research team. All data analysis procedures were conducted in Hebrew as the main language of participation in the study. During the first phase of analysis, team members independently read the transcripts, and a subset of 3 transcripts was coded in ATLAS.ti using a preliminary codebook to determine Intercoder Reliability (ICR) and codebook updates (43). In the second phase, the team reviewed and reflexively refined the preliminary codebook while jointly coding sample transcripts to resolve discrepancies and ensure consistency. Following this process, the revised codebook was applied to all transcripts. Coded sections were then compared within and across transcripts, and coded excerpts were organized into sub-themes and broader themes (40, 41). Drawing on the coded data, we iteratively developed an analytical framework to systematically identify and examine health-related climate risks, behaviors, and coping strategies and their related patterns. To enhance credibility, dependability, confirmability, and transferability of the analysis process, we developed an investigator triangulation strategy that included weekly peer debriefings and the exchange of reflexive memos and fieldnotes (44, 45).

## Findings

A total of 69 individuals from five localities in the Negev region took part in the study (see Table 1) in 10 focus group discussions (see Table 2). The sample included 48 (69.6%) women and 21 (30.4%) men, ranging in age from their early 20s to their mid-80s, with 58% of participants over the age of 60. Most participants reported having a below-average income (*n* = 43; 62.3%) or average income (*n* = 20; 29.0%). Finally, most participants (n = 38; 55.1%) did not indicate having any chronic illness or disability, 27 (39.1%) reported having a chronic illness, and four (5.8%) indicated having some disability. The final codebook was comprised of four categories including a total of 32 codes utilized in the analysis process (see Table 3).

**Table 1:** Focus group participant characteristics.

Characteristics	Count (*N* = 69)	%
Age		
20–29	4	5.8%
30–39	7	10.1%
40–49	13	18.8%
50–59	5	7.2%
60–69	15	21.7%
70–79	19	27.5%
80+	6	8.7%
Sex		
Female	48	69.6%
Male	21	30.4%
Medical background		
No background conditions	38	55.1%
Chronic illness	27	39.1%
Disability	4	5.8%
Living arrangement (*N* = 62)		
Alone	9	14.5%
With family	26	41.9%
With non-family	22	35.5%
With family or someone else	5	8.1%
Income		
Below average	43	62.3%
Around average	20	29.0%
Above average	6	8.7%
Mobility		
Car	45	65.2%
Public transport	33	47.8%
Taxi	2	2.9%
Walking	30	43.5%
Club car	7	10.1%

Living conditions for one focus group were not recorded (*N* = 62); Mobility: % calculated based on *N* = 69.

**Table 2:** Focus group characteristics.

Place	Number of participants	Defining characteristic
Bedouin town	7	Women only
Bedouin town	5	Men only
Unrecognized Bedouin village	7	Women only
Unrecognized Bedouin village	7	Men only
Small-size town (~500 residents)	6	Community resilience NGO employees and volunteers
Mid-size town (~40,000 residents)	8	Community resilience NGO employees and volunteers
Kibbutz	7	Older adults
City	9	Municipal enforcement and policing workers
City	6	Older adults
City	7	Immigrants

**Table 3:** Categories and codes used in the qualitative analysis.

Category	Codes
Knowledge of climate change risks	Populations vulnerable to climate change; Coping strategies; Manifestations of climate change; Personal experiences; General knowledge; Sources of knowledge; Health risks; General risks; Vulnerability to climate change
State/city/locality/community/health services	Responsibility of scale entities Barriers to action by scale entities Vulnerability at each scale; Activities/actions by scale entities; Scale-level solutions
Attitudes, awareness, and perceptions	Trust in authorities; Beliefs and climate change; Barriers to climate action; Perceived importance; Daily activities; Climate proactivity; Threat perception
Extreme weather events	Drought/prolonged dry periods; Heavy and/or prolonged rainfall; Increase in disease; Heat; Thunderstorms; Dust/sandstorms; Cold; Wind; Flooding; Frequency of extreme weather events

Four primary themes emerged from the focus groups: climate-related health impacts on vulnerable populations, the amplifying effects of structural barriers, community coping mechanisms, and trust in local and state authorities.

### Climate-related health impacts on vulnerable populations

Focus group participants identified a range of climate-related events highly prevalent in their area of living, and the impacts they had on their own health or the health of others. The health impacts associated with climate change were impacted, to a large degree, by the personal characteristics of the population. Focus group participants discussed a range of issues that they themselves or the people they knew experienced and that made them more vulnerable to climate-related health risks. These personal vulnerabilities echoed the common vulnerabilities in the literature and include pre-existing health conditions, young or older age, outdoor employment, lower income, and lack of access to a vehicle.

Experiencing pre-existing health conditions was described as a major factor that exacerbated the health impacts from climate change. Several participants shared how dust and sandstorms led to or exacerbated shortness of breath, pneumonia, and allergies among people with existing respiratory conditions (e.g., asthma). Similarly, extreme heat was described as having an adverse effect on the health and general well-being of participants with existing heart or vascular conditions or other pre-existing health issues. A female participant with multiple sclerosis explained the effect that heat had on her well-being:

*I have multiple sclerosis, and so the extreme heat paralyzes me … [I]f I was outside, even when I enter the house, it continues. It’s something [you can say] ‘OK I went to the AC – it will pass. No. It’s something that stays and bothers and hurts.* (Female, mid-size town).

Age, and its intersection with pre-existing health conditions, was also identified as representing a common vulnerability to climate-related health risks, especially in young children and older adults. Multiple participants described how children suffered from respiratory conditions due to dust and sandstorms. Other participants discussed children being vulnerable to heat strokes and the presence of venomous animals during hot weather when they played outdoors. These risks were more pronounced in the unrecognized Bedouin villages, which lack basic infrastructure such as being connected to the electric grid (and hence lack of air-conditioning units/ACs), paved roads (hence elevated dust), and waste treatment facilities (which attract venomous animals). As one female participant from an unrecognized Bedouin village shared:

*I have a granddaughter, [in] second grade. You see that when the weather changes, she immediately changes and starts coughing … Her health is dependent on the weather.* (woman, unrecognized Bedouin village).

Similar experiences were also shared regarding the older adults. Older participants described the difficulties of coping with extreme weather events, especially when coupled with pre-existing health conditions. For example, participants with high blood pressure or autoimmune diseases discussed the pain they experienced during hot weather. Other participants discussed how the desert dust and sandstorms caused shortness of breath, to the extent that people required oxygen masks. One female participant commented on the continued impact of dust and sandstorms on the older adults:

*Our sand … together with the dust and weather changes, have a strong effect on older adults and the older adults. Everyone has oxygen [masks], everyone has shortness of breath from all the dust…* (woman, unrecognized Bedouin village).

Climate change’s exacerbation of the health status of specific age groups and people with pre-existing health conditions often limits such people’s ability to perform outdoor activities, which contribute to physical, mental, and social health. Parents discussed stopping their children from spending time outdoors during extreme heat, sandstorms, or heavy rain. Participants also shared how they would cancel plans to meet family and friends, exercise, or even go to medical appointments during extreme weather events. Avoiding outdoor activities was mainly discussed as a protective measure; however, such avoidance could also have adverse health effects, as some participants explicitly or implicitly noted. The previous participant with multiple sclerosis further shared how she had to cancel a medical appointment due to extreme heat:

*I had to get to Soroka [the regional hospital]. It was a terribly hot day, and I had been waiting for this appointment for three months. I just called and said I could not leave the house in this heat.* (woman, mid-size town).

Yet for some, staying indoors was not an option. Individuals working outdoors have greater difficulty avoiding health risks related to climate change. Several participants discussed the impact that extreme heat had on the health of outdoor workers, including the risk of heat strokes, dizziness, and headaches. The participants of a focus group comprising municipal enforcement and police officers shared similar stories from their personal experiences. A female municipal enforcement and policing officer shared from her personal experience of working on a very hot day:

*I fainted. I felt dizzy*. Later she added that *if it’s very hot, it affects your health. Skin damage … No matter how much I protect myself from the sun with respect to long sleeves, a hat … I still have sunburns.* (woman, city).

The ability to protect oneself from climate-related health risks depends, to some degree, on the quality of equipment, infrastructure, and services that are available to the population. Inadequate home and public infrastructure and services may expose individuals to health risks or exacerbate outcomes. Thus, the structural barriers that different populations face are discussed in the next subsection.

### The amplifying effect of structural barriers

Alongside personal vulnerabilities, climate-related health effects are further amplified by structural barriers. For many individuals, lack of basic infrastructure and barriers to adequate services increase exposure to climate-related health risks and exacerbate them. These structural barriers were found to play out in several arenas, encompassing the home, the public realm and municipality, and the state. As one male participant from an unrecognized Bedouin village explained when asked about the effects of climate events on health:

*Even just simple dust with heat, together with the lack of basic conditions for living. I mean, at a minimum this could cause shortness of breath.* (man, unrecognized Bedouin village).

For vulnerable individuals, this lack of basic living conditions starts at home. Inadequate housing conditions expose residents to various climate-related health risks. Participants described how their homes were vulnerable to leaks and floods during heavy rains, which were linked to respiratory conditions and the risk of drowning. In unrecognized villages, homes are often just light tin structures, which lack proper insulation, amplifying both extreme cold and extreme heat. Multiple participants discussed the adverse health effects that such home environments have, especially on children and the older adults. In addition to physical health effects, concern over the ability of one’s home to withstand extreme weather was noted by some as a factor that increased stress. A female participant in a development town shared:

*Every time there is heavy rain, I feel stressed. A sort of trauma. This worry, like, “What’s going on in my house?” (Once) I got back from work and could not enter the house [because it was flooded].* (woman, mid-size town).

Similar concerns over flooding and inadequate infrastructure were also expressed with regard to the public realm. The failure of public spaces and services to function under extreme weather conditions is often attributed to inadequate treatment or maintenance by the local municipality. Several participants discussed the failure of their local governments to prevent roads from flooding or trees from falling down during heavy rain and strong winds, even as these events occur annually. A female participant from a mid-size town described the situation in her town:

*I live in one of these areas that supposedly underwent renovations. They replaced the roads, put up lights … everything looks nice. [But] during the first rain, you cannot leave the house. No drainage system! The entire street, from beginning to end, no drainage.* (woman, mid-size town).

The intersection of structural barriers with personal vulnerabilities is most apparent in the unrecognized Bedouin villages, where the state does not provide basic infrastructure or services, including street and pedestrian infrastructure, public services, electricity, fresh water, or sewage and waste treatment. Consequently, schools, healthcare facilities, transport, and other services are all located several kilometers outside the village. Multiple participants shared the difficulty of accessing services, especially during heavy rain, as traversing long distances by foot on unpaved roads poses various risks. One conversation among several male participants from an unrecognized village highlighted these issues:

*The school is far away from us, we are here, the people that live here. The school is a few kilometers away* (Participant 1). *No, it is difficult for them [the children] to go to school in the winter* (Participant 2). *There’s no paved road, and if you walk, you need to walk in the mud* (Participant 3). *There is a 70% absence rate. They do not go [to school] in the winter* (Participant 4). *During winter they do not go. They cannot get there* (Participant 1).

The absence of infrastructure and services also exposes residents to health risks close to their homes. Several participants from an unrecognized village, for example, linked the presence of mosquitoes, rodents, and venomous animals to the lack of adequate sewage and waste treatment. One mother from an unrecognized village described how the absence of waste and sewage facilities exposed children to various risks during out-of-school hours:

*They want to go out, the kids, the boys, they want to go. Where to? To the wadi* [canyon]*? It’s all dangerous. It’s full of garbage, there is a pool of stagnant water here and a sewage pool here.* (woman, unrecognized Bedouin village).

The structural barriers faced by each population and the inadequate treatment they received from local and national authorities eroded the belief that local or state actions could improve their living conditions. As a result, in many cases, participants felt a lack of trust, especially toward state intentions and actions. The void that the local and national governments left was often filled by everyday adaptation and through community support and engagement.

### Community as a coping mechanism

Study findings showed that participants relied heavily on local communities and their resources as a mechanism to cope with the effects of climate change. These support mechanisms operate during both routine and emergency times. However, throughout the study, the term community had multiple meanings that were highly contextual. Accordingly, whereas in some contexts participants defined their community as an extension of their family, or as their extended family, in other contexts the definition of community aligned with a physical location such as a neighborhood or a town. That said, participants throughout the study described their community as a key coping mechanism during routine times and emergencies.

Communities that provided both technical/physical and emotional support during routine times as well as during emergencies were described as reliable coping mechanisms. In the unrecognized villages, where the government did not provide electricity or running water, residents relied on generators and solar panels, which became major household expenses. Under these circumstances, support from the community even during routine times included, among other things, access to electricity. A female participant in a focus group in an unrecognized Bedouin village explained:

*To save on electricity, for example, when I married, I did not officially have my own electricity for two years. For two years I had a cable from my sister-in-law, from my husband’s brother. We connected a cable between the two homes… we saved money for two years until we had the money to install energy [solar panels].* (woman, unrecognized Bedouin village).

To this participant, and many others in her community, the extended family was the immediate support system. Faced by infrastructure limitations, and a lack of financial resources, resource-sharing within the extended family was described as a common practice. Similarly, participants described relying on the extended family when it came to the care of children and the older adults. In addition to the financial and technical support, emotional support was also described as being drawn from the community and from participation in communal care activities. And although in some cases the community was not necessarily the extended family, the relationships between community members were often described as resembling relationships in a family. A female participant in a mid-size town explained:

*The community helps us. It is because of our community organizer that I leave the house. It’s the volunteering that I do. It’s about being active…. it is [volunteering] like a family. If it wasn’t for this, why would I get out of bed?. I say the community does help; they make an effort* (woman, mid-size town).

During climate-related extreme weather events, participants noted that the same community mechanisms were used to provide physical and emotional support. These coping mechanisms built on extended families and close-knit neighborhood relationships. In the mid-size town, a participant recalled calling on her neighbors when her mother’s house was flooded:

*My mother’s house is built in such a way that it can be flooded… and she is an older adults woman. No one from the municipality helped me. We simply recruited all the neighbors… the municipality did not help; I could not tell them “Come to me” at all. I remember we went out and gathered our neighbors, all of us with rags during the floods and winds, and the men went up to put plastic sheets [on the roof].* (woman, mid-size town).

In an affluent small-size kibbutz, participants described having a community-owned internal network of generators that would spring into action as soon as the national electric network went down during a storm. The generator system was set up in this community following an extreme weather event, during which time the power shut down for a couple of days and all families and the older adults were evacuated to another kibbutz. Describing the community’s generator system, one of the participants explained:

*We have a generator for each neighborhood [in the kibbutz]. The moment the electricity goes down, the generator starts. It’s a matter of minutes, less than a minute*. (man, small-size kibbutz).

In both examples, one can see the internal communal mechanisms and resources, either human (family members and neighbors) or physical (generators), that became the community’s go-to coping mechanisms during emergencies. The role of the community as the most immediate coping mechanism in routine times and during emergencies hinges on intimate knowledge of the individuals in the community and their needs, as well as the structure of the community and its available resources. The focus on the community as a coping mechanism was evident in both the Jewish and Bedouin communities in this study. However, resources and infrastructures were much more limited for Bedouin communities who, in unrecognized villages, relied on communal networks for the provision of basic services such as electricity. Another marked difference between Bedouin and Jewish communities was the alignment between community and extended family in the Bedouin population.

However, in both the Bedouin and Jewish populations, the reliance on community coping mechanisms was based on the failure of the state and its institutions to take responsibility and provide appropriate solutions for the needs of its citizens. Participants in the study were not oblivious to the failures of the state. Addressing the question of responsibility, and the way it extended from the individual to the community and to the state, an older adults male participant living in a Bedouin town explained:

*Everyone is responsible. Every person is responsible for their own health, when he is sick, he needs someone to be with him. When he is young, he needs someone to take care of him. That means, you start with the head of the house until you get to the prime minister or head of state* (man, Bedouin town).

Thus, although participants described their communities as having a central role in caring for their members in general, and vulnerable members in particular, during both routine and emergency times, reliance on the community was also anchored in the gap between the community and the state and its institutions.

### Climate change experiences as rooted in lack of trust in the state

The study findings showed that the experience of climate change was rooted in a lack of trust in the state and its institutions. Members of vulnerable groups, ethnic minorities, or low socioeconomic groups often feel excluded by the state. In the case of the current study, lack of trust in the state and its institutions was expressed by both Bedouin and Jewish participants. However, this lack of trust was directed toward different agents of the state. Tensions exist between Bedouin residents and the state due to the historical background; since the early days of the Israeli state, there has been a continuous dispute over land rights and settlements of Bedouins in the Negev region (46). As a result, the Bedouin experience a lack of trust toward the state, and this lack of trust has been documented to impact issues surrounding health and healthcare (47, 48). Lack or no trust in the authorities was reported by all Bedouin participants even in the context of a conversation about the experience of climate change, as one participant noted:

*If people set up a good project, the government destroys it. A few years ago we built the mosque, and they came and destroyed it. The government destroyed the mosque. Every one of us who is sitting here has had their house destroyed twice or three times. The government comes and destroys his own house.*

Among most of the Bedouin focus groups, participants expressed negative emotions toward the authorities, especially the state, due to the feeling of severe neglect. In addition, many participants experienced this neglect as an intentional attempt to “make their life difficult” so that they would leave their villages, or make it easier for state authorities to evacuate them to other places, as one of the male participants living in an unrecognized village explained:

*This is only to make it difficult for the residents so that they will slowly leave the area, because in the future they want to evacuate this area. But they harass people more and make life even more difficult than it already is.* (man, unrecognized village).

A similar sentiment was expressed by a female participant:

*The State of Israel, who comes to the Divan* [the community’s meeting place] *and promises to help us if we vote for them in the elections. We are here for almost 25 years; we have been waiting since I got married 25 years ago. The day I got married, I lived in one room, a room and a toilet, [and I’m still in the same one room] and they say they will move us, that we are leaving. everyone is waiting [for this].* (woman, unrecognized village).

The disputed claim for land creates tension between the state and the residents and discourages Bedouins from moving from unrecognized villages to towns with higher access to infrastructure and services for fear that such a move might be interpreted as waiving their claim to the land.

The health crisis during the COVID-19 pandemic revealed the lack of coordination and the incompetence of the authorities, in addition to the lack of providing services that were appropriate and equal to the needs of different groups and populations. Multiple focus group participants across all populations pointed to the lack of health care during emergencies. However, among the Bedouin participants, the gaps were deeper, as they suffered from a lack of basic conditions such as housing, health services, and other services that were almost non-existent. In fact, in some ways they did not see the point of talking about climate change when they were still unrecognized and marginalized, and the impact of climate change on health was only one topic on their long list of concerns. As one participant explained:

*The story of medical neglect in the unrecognized villages is a matter in and of itself. We have many cases. It is true that we are all fated for death, but in many cases, death is mainly caused by the medical negligence of our clinic by the doctors in the clinic.* (man, unrecognized Bedouin village).

The severe infrastructure shortages in the Negev, especially in the unrecognized villages, such as the limited or non-existent water supply or lack of basic infrastructure, coupled with a strong sense of the state’s failure, left those who were most in need of medical services wanting, as one participant explained:

*You know that today we are in 2023 and there is a village in the State of Israel that is not recognized. Before we talk about the rise in global temperatures, I say let us talk about housing, about houses, about the basic things, and then we will move on to other things. As the saying goes, you cannot clap with one hand. We struggle alone.* (man, unrecognized Bedouin village).

Although lack of trust in the state and its institutions was central to the experiences of members of minority groups, participants belonging to the Jewish communities also experienced a lack of trust. In their case, however, the pathway of trust, or lack thereof, was more focused on the dysfunction of institutions such as municipalities, local authorities, and government ministries, as one participant explained:

*The local authority receives quite a lot of money. The local authority needs to take care of the health of the people. The Ministry of Health, I do not know anyone there with my eyes. I do know the local authority. Okay? I want to say to someone whom I know how to approach, My house looks like Balata Camp [a refugee camp near Nablus].* (woman, mid-size town).

Among the Jewish participants, trust in the local authority rather than in the state was the main issue. The Bedouin participants, for their part, put more trust in their family and community than in the state or local authorities, as one participant explained:

*In connection with the complaint, it [on named local street] has been known about for 20 years. But only after a man was killed, during heavy rains that flooded his whole house and yard, only then did they [the municipality] make the long overdue required change with the rainwater drainage.* (man, mid-sized town).

There were differences between the Jewish and Bedouin participants in their perception and experience of lack of trust toward the state. Among all the focus groups there was a lack of trust in the state, but among the Bedouin participants, the lack of trust was more profound and grounded in a historical and political background. All the participants in the Bedouin focus groups clearly indicated a lack of trust toward the state and the authorities. The Jewish participants, although they described the state as responsible in the context of climate change, their perceptions were more focused on accusations of incompetency, and a sense that the state and its institutions cannot be trusted to fulfil their duties.

## Discussion

Climate change does not affect human populations in a vacuum. As the findings of this study show, climate change intersects with other vulnerabilities to create a greater impact. To a large extent, climate effects are an extension of other existing inequalities intensified by exposure to extreme weather conditions. Climate hazards, therefore, pose a greater risk to populations that are both more vulnerable and more exposed, due to pre-existing social, economic, and health status. In the context of this study, various communities in the Negev region of Israel might be characterized differently based on socioeconomic status, ethnicity, and occupation as well as exposure and available infrastructures. Nevertheless, many similarities exist in terms of poverty and vulnerability as well as in how these populations utilize coping mechanisms to address the needs created by a changing climate.

From an individual perspective, study participants were concerned about the impact of climate change on their lives, and described these effects not only as stand-alone, but as an additional layer in the difficulty of managing their health, or as a struggle with failing infrastructures, whether physical or health system related. However, the extent of the impact was shaped by individuals’ and communities’ available resources, infrastructures, and capabilities. These findings align with prior research highlighting the social and individual factors that constrain climate change adaptation (18). Furthermore, recent evidence has emphasized that neither the impacts of climate change nor the options for adaptation are universal, and top-down adaptation strategies often fail to account for the complex interconnections between climate change and broader urban social, economic, political, and environmental vulnerabilities (49, 50). Notably, for individuals facing multiple overlapping vulnerabilities, climate change is often perceived as a lower-priority concern, as their immediate focus is directed toward navigating more urgent risks, unlike more privileged populations who can afford to prioritize climate-related issues.

Similarities between communities, as well as differences, are key to understanding climate change coping mechanisms. In all the populations addressed in this study, the community played a central role in caring for its members in general and its most vulnerable members in particular. The role of the community in caregiving was evident during routine times and emergencies as stated by the study participants. The role of the community as a caregiver often stemmed from intimate knowledge of the individual members of the community and their needs, as well as the structure of the community and its available resources. In addition to their role as providing support for community members, communities often served as mediators between individual members of the community, and their needs, and the state and its institutions – often by filling the gap created by the absence or incompetence of these national institutions. These dynamics align with research emphasizing the importance of social capital in fostering collective resilience, where bonding and bridging ties within communities enhances adaptive capacities even in vulnerable conditions (51, 52) and across diverse environmental (53) and sociopolitical challenges (54). Furthermore, these findings correspond with research suggesting that leveraging inherent community strengths while fostering connections with external actors and resources is essential for building both short- and long-term resilience (53).

Gaps between the state and its institutions and these community mechanisms and needs are often experienced as barriers to climate mitigation and adaptation interventions (55). These barriers were experienced by the study participants not only as infrastructure- and resource-based, but as barriers that were often rooted in the state’s and its institutions’ lack of knowledge regarding the needs of vulnerable individuals in the community, and sometimes even as deliberate neglect which resulted in a lack of trust in the state and its institutions. This lack of trust was also rooted in the large gaps in the provision of state services to the periphery compared to central Israel as part of a broader inequity that exists between central Israel and the periphery with respect to healthcare, employment opportunities, education, welfare, and transportation (6, 56).

These gaps between the state and its institutions and the community often follow known patterns of socioeconomic inequalities, making poor and marginalized populations more vulnerable to the effects of climate change (53, 57). In our study, unrecognized Bedouin villages already marginalized by the state, which does not provide full access to services and infrastructures as seen in other studies as well (58), were struggling to cope with the need to adapt to the effects of the changing climate with only very limited resources. Along the same lines, the residents of a mid-size town knew not to rely on the help of their weak municipality during an emergency. Thus, the already eroded trust between communities and the state is crucial to the understanding of climate change impact on communities and their respective coping mechanisms. Despite these challenges, our findings showed that these vulnerable groups displayed significant everyday adaptive behaviors, drawing on community resilience to support one another and developing innovative coping strategies. These patterns of bottom-up everyday adaptations by individuals and communities, documented elsewhere (16), take on local shape and character as they evolve in the context of other vulnerabilities. Although these grassroots mechanisms cannot and should not replace the role of the state, they represent a vital foundation for resilience that can be tapped into and enhanced through targeted policy interventions and resource support.

Although climate vulnerability is often associated with resource-limited countries, it is important to note that “islands” of climate vulnerability exist in high-income counties as well (5). These “islands” of climate vulnerability are often characterized by the same low-income characteristics such as poverty, limited access to infrastructures, and a contested relationship with local and national governments (59). Therefore, mapping out these high-risk areas of climate-related vulnerabilities becomes a priority in any climate adaptation strategy, and not just when low-income countries are concerned. The unique status and needs of various communities should be taken into account when designing climate intervention policies (49). However, such solutions should acknowledge existing gaps and vulnerabilities and build mechanisms to address these structural inequalities.

The findings of this study point to potential policy directions in developing strategies for interventions to support vulnerable communities coping with the effects of climate change. First and foremost, it is important to recognize that there are no “one-size-fits-all” solutions. Climate vulnerabilities, and coping mechanisms, are rooted in context (60). As such, strategies and solutions should be tailored to the needs, resources, and history of each community.

**Figure 2:**
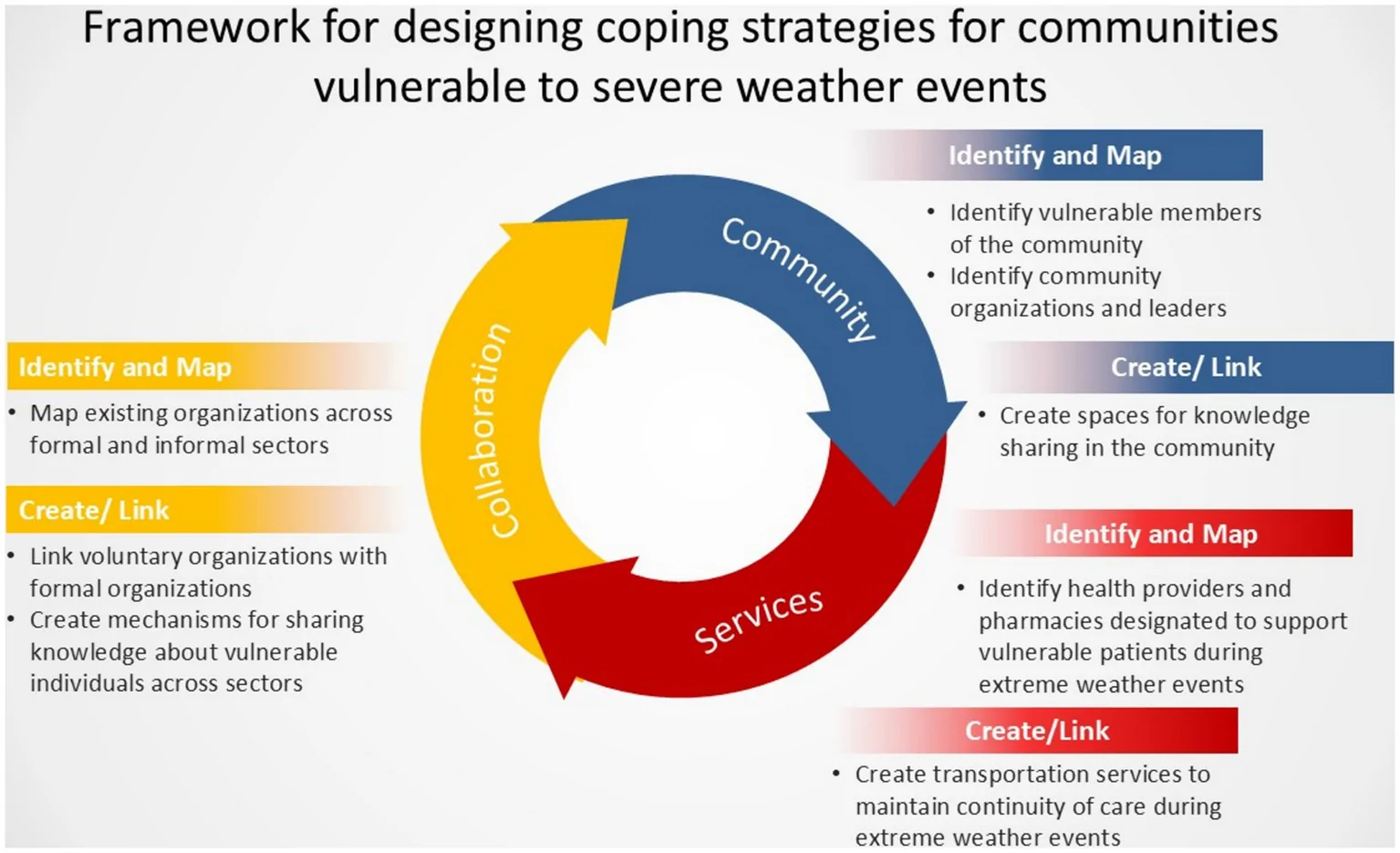
Framework for designing coping strategies for communities vulnerable to severe weather events.

That said, some overall guidelines would be helpful in designing coping strategies based on the principles of identifying and mapping needs and creating and linking opportunities and organizations to address these needs (see Figure 2). Fostering collaborations for knowledge exchange is essential for creating the appropriate solutions tailored for the needs of communities vulnerable to climate change. For example, collaborations must be built for the mapping of vulnerable populations and individuals across ministries and services, from welfare and health through education and religious and communal organizations. Such collaborations would include creating mechanisms for reaching out to people at risk and providing safety nets around vulnerable individuals (i.e., transportation services to maintain continuity of care during heat waves, pharmacies designated to support vulnerable patients). These strategies should also include identifying and strengthening community centers and religious organizations and community leaders as part of these streamlined community coalitions that do not operate only on a voluntary basis or during emergencies. In fact, building networks during routine times, and normalizing collaborations beyond organic structures, will allow for an easier mobilization of support during emergencies.

Although we mapped out climate-related health risks and behaviors among vulnerable populations in the Negev region of Israel, the current study has a few limitations. First, the study was conducted before the events of October 7, 2023 and thus does not address the war in the region nor its impact on the communities presented in this study. It is important to note that all of the communities participating in this study have been directly affected by the ongoing war, and future studies should assess the impact of the war on the ongoing vulnerabilities addressed in this study. Second, in order to address the needs of vulnerable populations in the study, we decided on a strategic oversampling of ethnic and political minorities, older adults participants and women acknowledging not only these groups’ overlapping vulnerabilities, and also their under-representation in climate research. Another important limitation stems from the chosen sample of communities. Although the sample of communities was chosen purposefully to represent the variety of populations and settlements in the Negev region, the sample was by no means a representative sample. Therefore, the transferability of the findings to other populations in Israel and elsewhere may be limited. Nevertheless, the study offers important insights that are potentially applicable to other settings as well. Along the same lines, participation in focus group discussions suffers from an unavoidable selection bias in terms of participants as well as limitations relating to focus-group dynamics and social desirability. To address these limitations, we extended our invitation to participate in the study among numerous populations and groups and worked to create various homogeneous groups facilitated by experienced researchers and supported by community leaders.

Societal systems of privilege and oppression are at the root of both vulnerability to climate change and social inequalities in health (61). It is the people with the least social and economic resources who are most exposed to the effects of climate change and who face the greatest difficulties in adapting to and recovering from these effects (57). The state’s obligation to provide its population with healthcare services is a central component of a broader worldview of social and climate justice.

## Conclusion

Coping with climate change ranges from the individual and the community to the state and its institutions. Vulnerable populations’ experiences with climate change are impacted by factors such as poverty, social marginality, violence, and barriers in accessing health and other services; climate change is not a stand-alone phenomenon. Therefore, climate change adaptation and mitigation cannot be distinguished from other challenges impacting the health and well-being of populations.

## Data Availability

The datasets presented in this article are not readily available because original source data includes focus group transcripts with participants that cannot be fully de-identified. Ethics approval does not include the publishing of private information. Requests to access the datasets should be directed to anatros@bgu.ac.il.
